# Genome-wide association study and candidate gene prioritization for stem architectural traits in Tartary buckwheat

**DOI:** 10.3389/fpls.2026.1960940

**Published:** 2026-10-30

**Authors:** Bingxin Zhai, Daowang Sun, Zihan Zhao, Jiaxing Xie, Ning Ma, Wenjie Lu, Guang Wang, Lihua Wang

**Affiliations:** Yunnan Provincial Key Laboratory of Agricultural Biotechnology, Key Laboratory of Southwestern Crop Gene Resources and Germplasm Innovation, Ministry of Agriculture and Rural Affairs, Biotechnology and Germplasm Resources Institute, Yunnan Academy of Agricultural Sciences, Kunming, China

**Keywords:** lodging resistance, mechanized harvesting, stem architecture, Tartary buckwheat, yield

## Abstract

Stem architecture is a key determinant of lodging resistance, yield potential, and mechanical harvestability in Tartary buckwheat (*Fagopyrum tataricum*), yet the genetic basis of stem-related traits remains poorly understood. In this study, a diverse panel of 244 Tartary buckwheat accessions was evaluated for five stem architectural traits, including plant height (PH), stem diameter (SD), branch number on the main stem (BNMS), node number on the main stem (NNMS), and stem wall thickness (TST), across three field environments (Huize 2025, Songming 2024, and Songming 2025). Genome-wide association study (GWAS) was performed using 280,377 high-quality Single Nucleotide Polymorphisms (SNPs) to identify loci associated with stem architecture traits. Substantial phenotypic variation was observed for all traits, with broad-sense heritability varying among traits and environments, likely reflecting the high sensitivity of Tartary buckwheat to environmental conditions across the three contrasting field environments. Strong positive correlations were detected between PH and NNMS, while SD and TST showed weak correlations with other traits, indicating partially independent genetic control. GWAS identified a major significant association region on chromosome (Chr) 4 affecting both PH and NNMS (Chr4: 31011474–31099420), while BNMS, SD, and TST displayed more dispersed or polygenic genetic architectures. Candidate genes within associated regions were mainly annotated to functions related to cell-wall modification, developmental regulation, and metabolic processes, suggesting potential mechanisms underlying stem architecture variation. Cross-trait analysis revealed extensive shared genetic control between PH and NNMS but relatively independent genetic regulation of BNMS and stem mechanical traits. These findings provide novel insights into the genetic architecture of stem traits in Tartary buckwheat and identify candidate loci for marker-assisted breeding of optimized plant architecture. Because PH and NNMS on the one hand and BNMS, SD and TST on the other are under largely independent genetic control, the Chr4 block and the trait-specific loci identified here can be combined by marker-assisted pyramiding to develop lodging-resistant, mechanization-ready cultivars.

## 1 Introduction

Tartary buckwheat (*Fagopyrum tataricum* Gaertn.) is a pseudocereal crop belonging to the Polygonaceae family. It is believed to have originated in southwestern China and has long been cultivated in mountainous regions of China and the Himalayas, with additional cultivation reported in parts of Europe and other temperate regions (Zhang et al., 2017; Hunt et al., 2018). China is the largest producer of Tartary buckwheat, with an estimated harvested area of approximately 300,000 ha and an annual output of 300,000–500,000 t, most of it grown at high elevation in Yunnan, Sichuan and Guizhou, which together account for about 80% of the national growing area. Average grain yield, however, remains low, generally between 0.9 and 2.25 t ha^-^¹, far below that of the major cereals (Saunders Bulan et al., 2017). The crop has attracted attention because of its nutritional value, especially its high levels of rutin and other flavonoids with antioxidant, anti-inflammatory, and antidiabetic activities (Zhu, 2016; He et al., 2026). However, susceptibility to lodging and loose plant architecture severely constrain the yield and mechanized harvesting of Tartary buckwheat, and its grain yield remains far below that of the major cereals (Xiang et al., 2016, 2019; Morishita et al., 2020). Therefore, it is necessary to shape an ideal plant architecture through genetic improvement to increase buckwheat yield and adapt to mechanized harvesting.

Plant architecture refers to the spatial organization of plant organs, including stems, branches, and leaves, which strongly affects canopy light interception, lodging resistance, planting density, and harvesting efficiency (Wang and Li, 2008; Wang et al., 2018). In cereals, the genetic control of plant and stem architecture has been dissected in considerable detail. A recent genome-wide association studies (GWAS) of the Sorghum Association Panel, which evaluated 14 plant architectural and 10 biomass-yield traits over two seasons, detected 321 significant SNPs and 22 pleiotropic loci, and identified three genomic hotspots on chromosomes 6, 7 and 9 that, together with the classical dwarfing genes Dw1, Dw2 and Dw3, control plant height, stem diameter and stem volume (Singh et al., 2025). Notably, several sorghum candidates encode cell-wall-modifying enzymes, including an endoglucanase and a pectinesterase at the season-stable pleiotropic locus S03_15463061 and an invertase/pectin methylesterase at a tiller-number locus, indicating that cell-wall remodeling and hormone signaling are recurrent themes in the genetic control of stem architecture. Plant architecture traits, such as the node number on the main stem (NNMS), branch number on the main stem (BNMS), stem diameter (SD), and stem wall thickness (TST), are closely related to canopy structure, light interception, lodging, and yield (Sun et al., 2021; Yang et al., 2023). Taller plants with more nodes may have more reproductive sites, but they are also more likely to lodge. In contrast, thicker stems and thicker stem walls can provide stronger mechanical support. Branch number may influence the allocation of assimilates between vegetative and reproductive growth (Xiang et al., 2019; Yang et al., 2023). Improving the combination of these traits is therefore important for Tartary buckwheat breeding, but the genetic mechanisms underlying them are still not well understood. Whether the genetic modules identified in cereals are conserved in this dicot pseudocereal remains unknown.

The publication of the Tartary buckwheat reference genome (Zhang et al., 2017) and the accumulation of resequencing data from diverse germplasm collections have facilitated the application of GWAS in this species (Zhang et al., 2021a; He et al., 2024a). Previous studies have used GWAS to dissect the genetic basis of agronomic performance, abiotic stress tolerance, and metabolic traits in Tartary buckwheat, thereby identifying multiple associated loci and candidate genes. These traits included dehulling efficiency (Yang et al., 2024b), grain weight (Zhang et al., 2021a), drought (He et al., 2024b), and salt tolerance (Lu et al., 2025), flavonoid accumulation (Zhang et al., 2021a), ionomic variation (Yin et al., 2020; Wang et al., 2025b), and rutin biosynthesis (Mao et al., 2025). However, the genetic basis controlling the stem structural characteristics of Tartary buckwheat remains poorly understood. Current studies have shown that among plant height (PH), NNMS, BNMS, PH generally exhibited relatively high heritability (Li et al., 2012; Xiang et al., 2019; Hou et al., 2022). In addition, selective-sweep and association analyses identified six SNP loci associated with PH and highlighted three genes with distinct predicted functions: FtPinG0505903200, a protein kinase belonging to the leucine-rich repeat receptor-like kinase (LRR-RLK) family whose Arabidopsis homolog *AtVRLK1* regulates the switch between cell elongation and secondary cell-wall thickening; FtPinG0606457100, a translation factor whose Arabidopsis ortholog plays a crucial role in plant growth; and *FtSKIP14* (FtPinG0009116500.01), an F-box protein that forms part of the SCF E3 ubiquitin-ligase complex, is approximately 85% identical to Arabidopsis *SLY1*, and mediates gibberellin signaling through ubiquitin-dependent degradation of DELLA proteins, a Ser-to-Asn substitution in its GGL domain underlying the gibberellin-deficient dwarf mutant ftdm (Sun et al., 2021; Zhang et al., 2021a). Therefore, systematic research on the genetic basis of stem structural characteristics in Tartary buckwheat is an important foundation for advancing its breeding process.

In this study, we performed a multi-environment GWAS to investigate five stem architecture traits in Tartary buckwheat, including PH, NNMS, BNMS, SD, and TST. The analysis was based on 244 diverse accessions evaluated in three field environments, together with best linear unbiased estimates (BLUE) calculated across environments. We used 280,377 high-quality SNPs from whole-genome resequencing and applied a linear regression model to detect trait-associated loci. Candidate genes were then prioritized using GWAS signals, functional annotation, model-species homology, and evidence from related traits. The main objective was to better understand the genetic basis of stem architecture variation and to provide candidate loci for future breeding of Tartary buckwheat. We show that (i) a single major region on Chr4 (31.01–31.10 Mb) is associated with both PH and NNMS and contains candidate genes potentially involved in cell-wall remodeling and transcriptional regulation; (ii) BNMS, SD and TST are controlled by largely independent and more polygenic loci; and (iii) the same classes of cell-wall- and hormone-related genes that determine stem architecture in cereals are also implicated in Tartary buckwheat. Because these trait groups are largely genetically independent, PH, canopy branching and stem mechanical strength can be improved simultaneously without antagonistic trade-offs, and the Chr4 block provides an immediately usable target for marker-assisted selection.

## 2 Materials and methods

### 2.1 Plant materials and field experiments

A total of 244 Tartary buckwheat accessions were used in this study. These materials originated from Yunnan, Sichuan, and Guizhou Provinces, China, and are currently preserved at the Yunnan Provincial Repository for Crop Germplasm Resources (Kunming, Yunnan, China). The accessions analyzed here were retained from a previously reported 313-accession Tartary buckwheat germplasm panel described by (Zhai et al., 2026). In the present study, plant architecture-related traits were newly evaluated under multi-environment field conditions, and accessions with complete phenotypic records and high-quality resequencing data were retained for subsequent analyses. Detailed information for all accessions, including accession ID, geographic origin, latitude, and longitude, is provided in **Supplementary Table S1**.

Field experiments were conducted in three environments, including Songming in 2024 (24SM), Songming in 2025 (25SM), and Huize in 2025 (25HZ). The Songming experimental site was located at the experimental station of the Yunnan Academy of Agricultural Sciences in Xiaojie Town, Songming County, Kunming City, Yunnan Province (25°21′23″N, 103°06′39″E), whereas the Huize experimental site was located in Jiache Town, Huize County, Qujing City, Yunnan Province (25°59′17″N, 103°24′44″E). All field trials were arranged using a randomized complete block design with three biological replicates. Each accession was planted in a single-row plot 2 m in length, with 20 cm between rows and 15 cm between plants. Standard agronomic management practices were applied consistently across all environments during the growing season. At maturity, five plants with normal growth were randomly selected from each plot for trait evaluation, and the mean value of the five plants was used for subsequent analyses. Young leaves were collected before flowering at the Songming experimental site, immediately frozen in liquid nitrogen, and stored at −80 °C for DNA extraction and resequencing. The three environments differed in altitude, growing-season temperature and rainfall (**Table 1**).

**Table 1:** Environmental characteristics of the three field trial environments.

Environment	Code	Location	Altitude (m)	Mean temperature (°C)	Total precipitation (mm)
Huize 2025	25HZ	Jiache Town, Huize County, Qujing (25°59′17″N, 103°24′44″E)	2501.0	15.25	1324.3
Songming 2024	24SM	Xiaojie Town, Songming County, Kunming (25°21′23″N, 103°06′39″E)	1914.0	18.05	1153.4
Songming 2025	25SM	Xiaojie Town, Songming County, Kunming (25°21′23″N, 103°06′39″E)	1914.0	17.50	1393.8

Geographic coordinates are given in decimal degrees (WGS84). Mean temperature and total precipitation represent the growing season from June to October of each experimental year and were derived from the ERA5-Land reanalysis dataset. Mean temperature was calculated as the average 2-m air temperature, and total precipitation was calculated as the cumulative precipitation during the same period. Altitude refers to the elevation of the experimental site above mean sea level.

### 2.2 Phenotypic trait measurement

Five plant architecture-related traits were evaluated in this study, including PH, SD, BNMS, NNMS, and TST. Trait measurements were performed at the mature stage in all three environments. PH was measured as the distance from the stem base to the tip of the longest stem branch. NNMS was recorded as the total number of nodes from the stem base to the apex of the main stem, whereas BNMS represented the number of primary branches originating from the main stem. SD and TST were measured at the middle position between the first and second internodes using a digital caliper. Phenotypic data were further integrated using best linear unbiased estimation (BLUE) values for downstream statistical analysis and GWAS.

### 2.3 Statistical analysis of phenotypic data

Descriptive statistical analyses of phenotypic data across different environments were performed using Python scripts, including mean, median, standard deviation, coefficient of variation (CV), skewness, kurtosis, and phenotypic variation range. Histograms combined with fitted normal distribution curves were generated to evaluate the distribution patterns of different traits. Pearson correlation analysis was conducted to assess the relationships among traits in different environments, and correlation heatmaps were generated using Python. Broad-sense heritability (H²) was estimated using a leave-one-out approach based on variance decomposition across field trial environments. For each environment-specific correlation plot, H² for a given trait was estimated by excluding that environment and using the remaining non-BLUE environments. Specifically, H² was calculated as:


$$H^{2}=\sigma^{2}g/\left(\sigma^{2}g+\sigma^{2}e\right)$$

where σ²g is the among-genotype variance estimated from the genotypic means across the retained environments, and σ²e is the mean within-genotype variance across environments (i.e., the average of per-genotype variances across the retained environments). For the BLUE-based correlation plot, all three field trial environments (25HZ, 24SM, and 25SM) were used. BLUE for each accession were obtained from a mixed model in which genotype was treated as a fixed effect and environment, replication within environment, and the genotype × environment interaction were treated as random effects; the BLUE values were used as the multi-environment phenotype for correlation analysis and GWAS. Pearson correlation coefficients were calculated for all pairwise trait combinations within each environment using pandas. DataFrame.corr(), and statistical significance was assessed using Fisher’s z-transformation with a two-tailed test. All phenotypic analyses were performed in Python (version 3.13.5, Anaconda distribution) using the following packages: pandas (version 2.3.3; McKinney, 2010), numpy (version 2.3.5; Harris et al., 2020), scipy (version 1.17.1; Virtanen et al., 2020), and matplotlib (version 3.10.8; Hunter, 2007).

### 2.4 DNA extraction and whole-genome resequencing

Young leaves collected from each accession were used for genomic DNA extraction. DNA was extracted using a NucleoMag Plant DNA Extraction Kit (Takara, USA) according to the manufacturer’s instructions. DNA quality and concentration were evaluated using a NanoDrop 8000 spectrophotometer (Thermo Scientific, USA), and DNA integrity was further checked by 1% agarose gel electrophoresis. Qualified DNA samples were used for whole-genome resequencing library construction. Genomic DNA was fragmented using the MGIEasy Fast Enzyme Digestion Library Preparation Reagent Set V2.0 (MGI, Shenzhen, China), and libraries with an average insert size of approximately 350 bp were prepared following the standard protocol provided by the manufacturer. The libraries were subjected to paired-end sequencing (PE150) on the MGI DNBSEQ-T7 platform by Huazhi Biotechnology Co., Ltd. (Changsha, China). Raw sequencing data generated in this study were used for subsequent SNP detection and genome-wide association analyses.

### 2.5 Read alignment, SNP calling, and variant filtering

Raw sequencing reads were first processed to remove adapter sequences, low-quality reads, and reads containing excessive ambiguous bases using FASTP software (Chen et al., 2018). Clean reads were then aligned to the Tartary buckwheat reference genome (GCA_002319775.1) using Sentieon based on the BWA-MEM algorithm (Li, 2013). Sequencing coverage and mapping statistics were calculated from the alignment results using SAMtools (Li et al., 2009).

Variant detection was performed using the HaplotypeCaller module in GATK (McKenna et al., 2010). SNPs were further filtered to obtain a high-quality variant dataset for downstream analyses. Variants with low sequencing quality or unreliable mapping signals were removed based on the following criteria: QD < 2.0, MQ < 40.0, FS > 60.0, SOR > 4.0, MQRankSum < −12.5, ReadPosRankSum < −8.0, or low minor allele frequency (MAF). After filtering, a total of 280,377 high-quality SNPs were retained for subsequent population genetic analysis and genome-wide association analysis.

### 2.6 Genome-wide SNP distribution and functional annotation

Genome-wide SNP density was calculated using VCFtools with a sliding window size of 1 Mb (Danecek et al., 2011). SNP density distribution across the eight chromosomes was visualized using R scripts based on the physical positions of SNPs on the Tartary buckwheat reference genome.

Functional annotation of SNPs was performed according to the genomic annotation of the reference genome. SNPs were classified into exon, intron, upstream, downstream, and intergenic regions based on their physical locations. Upstream and downstream regions were defined as the 2-kb regions adjacent to transcription start sites and transcription termination sites, respectively. Intronic regions were generated by subtracting exon regions from annotated gene regions using BEDTools (Quinlan and Hall, 2010). To avoid multiple assignments, SNPs were categorized into mutually exclusive classes following the priority order: exon > intron > upstream > downstream > intergenic. The numbers and proportions of SNPs in different genomic regions were subsequently calculated.

### 2.7 Genome-wide association analysis and candidate gene annotation

GWAS was performed for five plant architecture-related traits, including PH, SD, BNMS, NNMS, and TST. Association analysis was conducted separately for each environment as well as for the multi-environment BLUE values. GWAS was performed using the linear regression model (--linear: additive allelic effect, no additional covariates) implemented in PLINK v1.9 (Purcell et al., 2007). Manhattan plots and quantile–quantile (Q–Q) plots were generated using Python scripts, and the genomic inflation factor (λGC), defined as the ratio of the median observed to the median expected χ² statistic, was calculated for each trait–environment combination. SNPs with P values lower than 1 × 10–^5^ were considered significant association signals. This threshold was adopted as an exploratory cut-off that balances type I and type II error in a panel of moderate size: a strict Bonferroni correction for 280,377 tests would require P < 1.8 × 10^-7^, which is overly conservative for 244 accessions and would exclude genuine loci of moderate effect. Confidence in the reported loci therefore rests not on the nominal threshold alone but on three additional criteria: recurrence of the same locus in independent environments and in the multi-environment BLUE dataset, co-localization with phenotypically correlated traits, and the magnitude of the signal.

Candidate genes were identified within a ±200 kb window surrounding significant SNP loci based on the Tartary buckwheat reference genome annotation. This interval was selected according to previous GWAS studies in Tartary buckwheat, in which a ±200 kb window around lead SNPs was used for candidate gene identification (Zhang et al., 2021a). Functional annotation of candidate genes was performed using multiple annotation resources, including Pfam domains, Gene Ontology (GO), and InterPro annotations from the Tartary buckwheat reference genome database. To further infer gene functions, homologous genes in *Arabidopsis thaliana* (TAIR10) and *Oryza sativa* (IRGSP-1.0) were identified using BLASTp searches (Altschul et al., 1997). The best hit for each query sequence was retained, and homologous relationships were defined using sequence identity, query coverage, and E-value thresholds. Candidate genes were subsequently prioritized using a five-dimensional composite scoring framework that integrated GWAS statistical significance, cross-species homology support, functional relevance, annotation completeness, and the number of supporting SNPs, as described below.

For each candidate gene, a composite score (ranging from 0 to 1) was calculated as the weighted sum of five criteria. (1) Statistical significance (weight = 0.30): the −log_10_(*P*) value of the lead SNP was normalized against the 95th percentile of all lead SNP −log_10_(*P*) values across the full dataset and clipped to a maximum of 1.0. (2) Cross-species homology support (weight = 0.25): genes with high-confidence homologs (sequence identity ≥ 70% and query coverage ≥ 70%) in both *A. thaliana* and *O. sativa* received a score of 1.0; genes with a high-confidence hit in only one species received 0.5; genes with lower-confidence hits in one or both species received partial scores of 0.25 per species; genes with no detectable homolog received 0. (3) Functional relevance (weight = 0.20): genes assigned to agronomically relevant functional categories — including transcription factor families (bZIP, MYB, bHLH, WRKY, NAC, AP2/ERF, HD-ZIP, PLATZ, TCP, YABBY, GATA, and zinc-finger proteins), hormone signaling pathways (auxin, gibberellin, cytokinin, brassinosteroid, ethylene, abscisic acid, jasmonate, and salicylate), cell wall biosynthesis and remodeling (polygalacturonase, cellulose synthase, expansin, pectin methylesterase, and XTH), receptor-like kinases (RLK/LRR-RLK), transporters (ABC transporters), and core developmental regulators (flowering time and cell cycle) — received a score of 1.0; genes with other known functional annotations received 0.4; genes with no functional annotation received 0. (4) Annotation completeness (weight = 0.15): the availability of Pfam domain IDs, InterPro entry IDs, and GO terms each contributed equally (1/3) to this dimension. (5) Number of supporting SNPs (weight = 0.10): the count of significant SNPs within the same linkage disequilibrium (LD) block co-annotated to the candidate gene was log_2_-transformed and normalized to the maximum value observed across all candidates.

Candidate genes were classified into three priority tiers based on their composite scores. Tier 1 (Core) comprised genes with a composite score at or above the 85th percentile of all candidates that additionally possessed either a high-relevance functional annotation or full cross-species homology support; these represent the most confidently prioritized candidates. Tier 2 (Supporting) comprised genes with a composite score at or above the 60th percentile. Tier 3 (Additional) comprised the remaining annotated candidates. For each of the five architectural traits examined — PH, SD, BNMS, NNMS, and TST — candidate genes were ranked by composite score within each trait independently.

## 3 Results

### 3.1 Phenotypic variation, distribution, and correlation analysis

Descriptive statistical analysis was performed across various environments (25HZ, 24SM, 25SM, and BLUE) to assess the phenotypic performance of the traits studied (**Table 2**). All investigated traits, including PH, SD, BNMS, NNMS, and TST, exhibited substantial phenotypic variation within the population. The mean values of PH ranged from 95.65 cm to 175.92 cm, while SD varied from 6.95 mm to 11.57 mm across environments. Similarly, BNMS and NNMS also showed broad variation, with mean values ranging from 6.07 to 7.61 and 13.11 to 24.36, respectively. The coefficients of variation (CV) ranged from 0.05 to 0.23, indicating moderate phenotypic diversity among traits, with TST showing relatively higher variability compared to other traits.

**Table 2:** Summary statistics of phenotypic traits across multiple environments.

Trait	Env	Max	Min	Mean	IQR	StDev	CV	Skewness	Kurtosis	Fold change	H^2^
PH	25HZ	158.60	88.00	123.97	17.30	12.97	0.10	-0.36	3.13	1.80	0.03
	24SM	204.93	145.03	175.92	14.06	11.67	0.07	-0.23	2.99	1.41	0.18
	25SM	133.05	54.40	95.65	21.16	15.34	0.16	-0.11	2.75	2.45	0.05
	BLUE	160.82	120.21	142.91	10.74	8.35	0.06	-0.37	2.93	1.34	0.04
SD	25HZ	9.82	5.76	7.81	1.11	0.82	0.11	-0.21	2.59	1.70	0.03
	24SM	13.66	9.44	11.57	1.06	0.84	0.07	0.14	2.90	1.45	0.30
	25SM	9.76	4.24	6.95	1.27	1.05	0.15	-0.08	2.99	2.30	0.04
	BLUE	10.99	8.27	9.48	0.71	0.53	0.06	0.13	2.83	1.33	0.04
BNMS	25HZ	8.20	4.00	6.07	1.10	0.86	0.14	-0.06	2.89	2.05	0.30
	24SM	9.72	4.69	7.14	1.18	0.98	0.14	0.03	2.99	2.07	0.21
	25SM	10.00	5.20	7.61	1.25	0.92	0.12	-0.28	2.91	1.92	0.23
	BLUE	8.29	5.41	6.99	0.77	0.56	0.08	-0.14	2.70	1.53	0.18
NNMS	25HZ	16.60	10.25	13.11	1.49	1.21	0.09	0.11	3.23	1.62	0.05
	24SM	28.46	20.20	24.36	1.92	1.47	0.06	-0.21	2.98	1.41	0.15
	25SM	22.50	11.25	17.12	2.87	1.99	0.12	-0.28	2.82	2.00	0.02
	BLUE	22.32	17.02	19.74	1.42	1.03	0.05	-0.12	2.73	1.31	0.03
TST	25HZ	2.57	1.15	1.85	0.37	0.29	0.16	0.05	2.75	2.24	0.16
	24SM	4.09	2.08	3.02	0.50	0.40	0.13	0.32	2.87	1.97	0.26
	25SM	3.64	1.00	2.29	0.69	0.53	0.23	0.26	2.95	3.63	0.07
	BLUE	3.43	1.96	2.55	0.31	0.23	0.09	0.19	3.38	1.76	0.09

PH, plant height (cm), measured as the distance from the stem base to the tip of the longest stem branch; SD, stem diameter (mm), measured at the middle between the first and second internodes using a digital caliper; BNMS, branch number on the main stem (count), representing the number of primary branches originating from the main stem; NNMS, node number on the main stem (count), defined as the total number of nodes from the stem base to the apex of the main stem; TST, stem wall thickness (mm), measured at the middle between the first and second internodes. Env indicates the environment, including 25HZ, 24SM, 25SM, and BLUE. Max, maximum value; Min, minimum value; Mean, average value; IQR, interquartile range; StDev, standard deviation; CV, coefficient of variation; Skewness, skewness coefficient; Kurtosis, kurtosis coefficient; Fold change, ratio of maximum to minimum values; H², broad-sense heritability estimated using variance decomposition across environments.

The fold change values further confirmed the existence of considerable variation among individuals, particularly for PH and TST, suggesting that these traits are highly variable and suitable for genome-wide association analysis. Skewness and kurtosis values of most traits were close to zero and three (**Table 2**), respectively, indicating that the phenotypic distributions approximated normal distributions across environments (**Figure 1**). This continuous and near-normal distribution pattern suggested that these traits are typical quantitative traits controlled by multiple genes. The broad-sense heritability (H²) estimates varied among traits and environments (**Table 2**). BNMS showed relatively higher heritability (H² = 0.18–0.30), while PH, SD, NNMS, and TST exhibited lower heritability, with some values as low as 0.02. The three experimental environments varied in altitude (1,914–2,501 m a.s.l.), mean growing-season temperature (15.25–18.05°C), and rainfall conditions (**Table 1**), which likely contributed to pronounced genotype × environment interactions and consequently lower H² estimates for some traits, particularly those more sensitive to environmental variation (Li et al., 2021).

**Figure 1:**
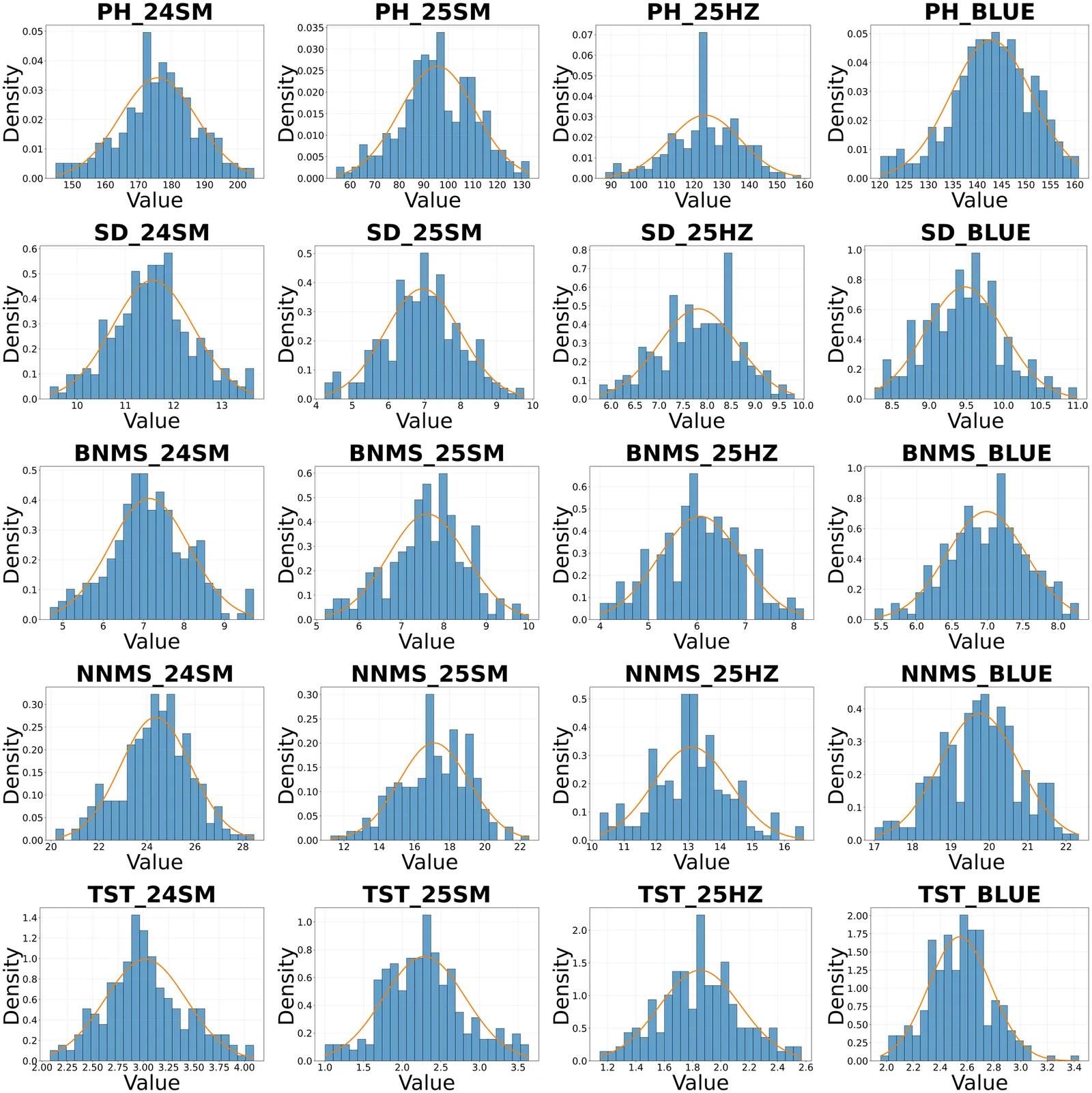
Frequency distributions of stem architectural traits based on observed phenotypic values and multi-environment BLUE estimates. PH, plant height; SD, stem diameter; BNMS, branch number on the main stem; NNMS, node number on the main stem; TST, stem wall thickness. 24SM, Songming 2024; 25SM, Songming 2025; 25HZ, Huize 2025; BLUE, best linear unbiased estimate calculated across multiple environments.

Correlation analysis was performed to evaluate the relationships among traits under different environments (**Figure 2**). Pearson’s correlation coefficients (upper triangle) revealed that PH showed a consistently significant positive correlation with SD and NNMS across all environments. SD also exhibited significant positive correlations with NNMS across all environments, suggesting a coordinated relationship between these traits. Genetic correlation coefficients (lower triangle) showed similar trends to phenotypic correlations, indicating that the observed relationships among traits are largely genetically controlled. Notably, strong genetic correlations were observed between PH and NNMS, and between SD and NNMS, suggesting potential shared genetic bases or pleiotropic effects of underlying loci.

**Figure 2:**
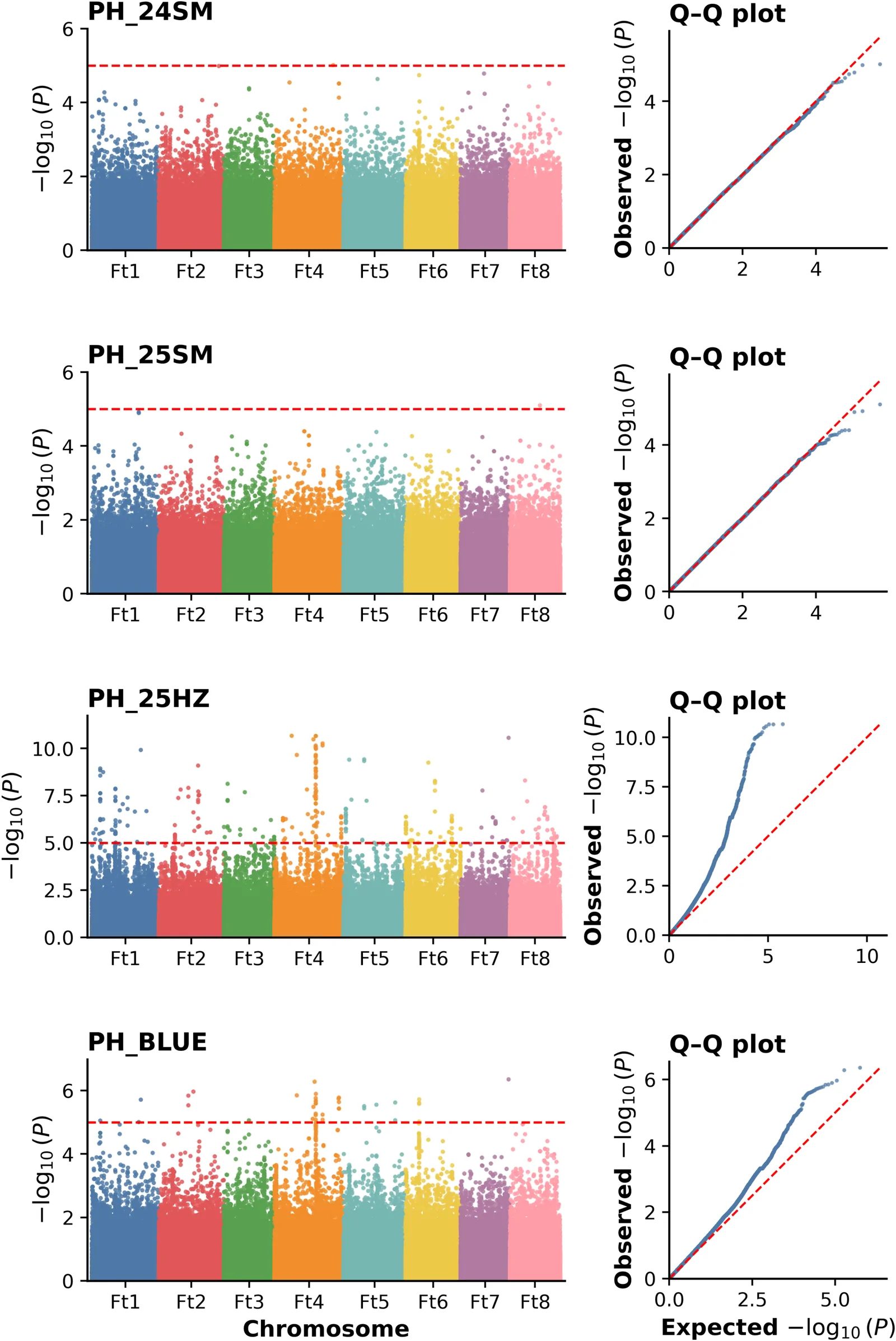
Correlation analysis of stem architectural traits using environment-specific phenotypic values and multi-environment BLUE estimates. The upper triangle shows phenotypic correlations based on observed accession means and the lower triangle shows genetic correlations. The BLUE panel was calculated using best linear unbiased estimates across environments. PH, plant height; SD, stem diameter; BNMS, branch number on the main stem; NNMS, node number on the main stem; TST, stem wall thickness. 24SM, Songming 2024; 25SM, Songming 2025; 25HZ, Huize 2025; BLUE, best linear unbiased estimate.

Overall, the substantial phenotypic variation, approximately normal distribution, significant trait correlations, and moderate heritability collectively demonstrated that the study population is suitable for subsequent GWAS and candidate gene identification.

### 3.2 Genome-wide distribution and functional annotation of SNPs

We characterized the genome-wide distribution of SNPs (**Figure 3**). Along the physical positions, most chromosomes exhibited a consistent distribution pattern, with higher SNP density in the central regions and relatively lower density toward both chromosomal ends. Specifically, SNP density gradually increased from the termini toward the middle, forming continuous high-density regions in the central parts, followed by a decrease toward the distal ends. This “low–high–low” pattern was particularly evident in chromosomes 2, 3, and 7, suggesting a relatively conserved distribution structure across chromosomes. In addition, distinct local enrichment peaks were observed on certain chromosomes. These regions exhibited markedly higher SNP density than adjacent regions, forming discrete peak-like patterns.

**Figure 3:**
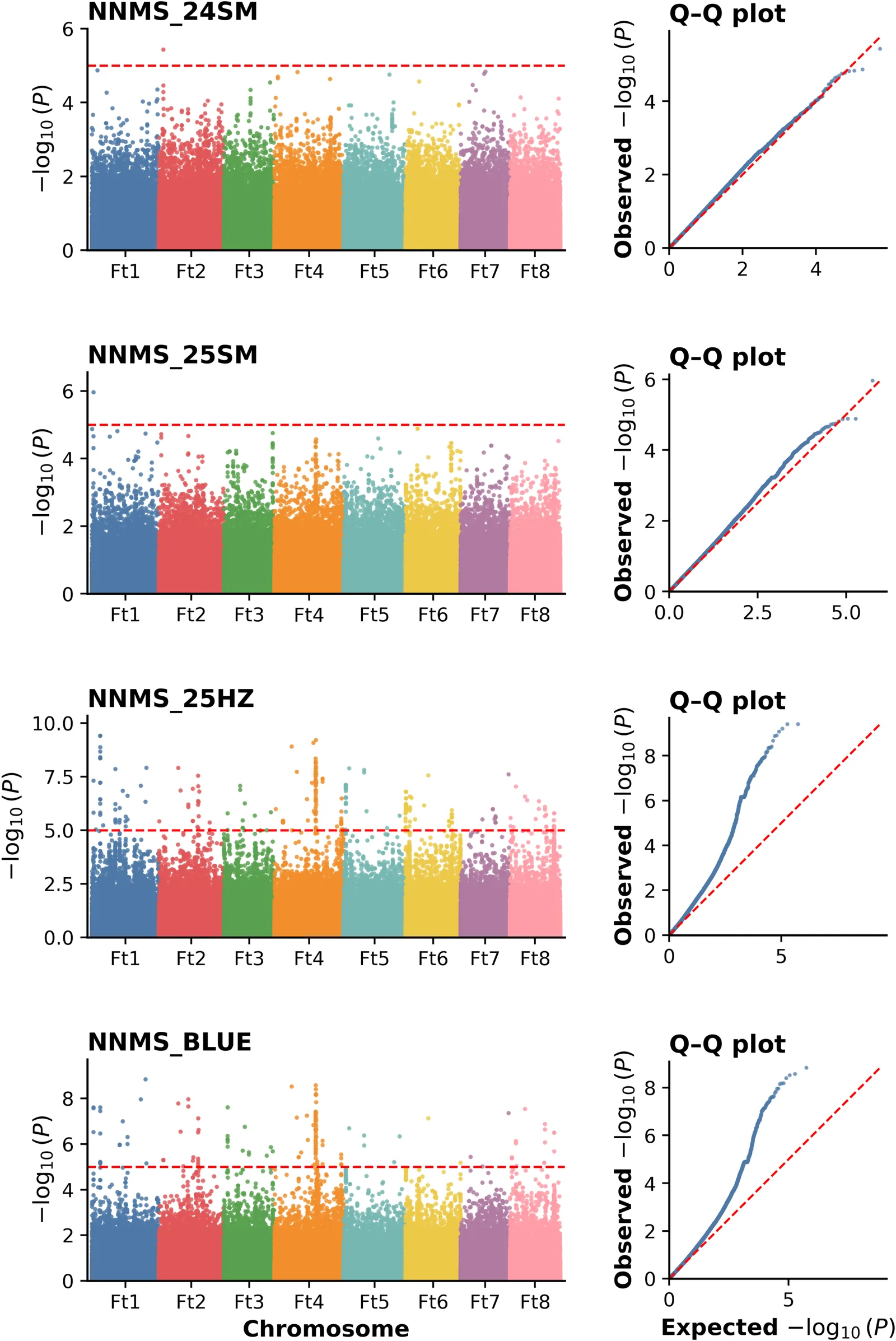
Genome-wide distribution of SNP density across eight chromosomes.

Functional annotation indicated that the majority of SNPs were located in intergenic regions, accounting for 236,767 SNPs (84.45%) of the total (**Table 3**). Upstream and downstream regions contained 15,879 (5.67%) and 11,620 (4.14%) SNPs, respectively. In contrast, only 8,308 SNPs (2.96%) were located in exonic regions, and 7,803 SNPs (2.78%) were distributed within intronic regions, while no SNPs were identified in splice site regions.

**Table 3:** Functional annotation distribution of genome-wide SNPs based on gene structure regions.

Type	Count	Ratio (%)
Downstream	11620	4.14
Exon	8308	2.96
Intergenic	236767	84.45
Intron	7803	2.78
Upstream	15879	5.67
Total SNPs	280377	100

Genome-wide SNPs were functionally annotated based on the reference genome annotation. SNPs were categorized into exon, intron, upstream, downstream, and intergenic regions. Upstream and downstream regions were defined as 2 kb from transcription start and termination sites, respectively. Intronic regions were inferred as gene regions excluding annotated exons. To avoid multiple assignments, SNPs were classified into mutually exclusive categories using a hierarchical scheme (exon > intron > upstream > downstream > intergenic). The total number of SNPs and their relative proportions are presented for each category.

Overall, these results indicate that most genetic variations are distributed in non-coding regions, whereas variants located in coding and regulatory regions represent a smaller proportion, consistent with the conserved nature of functional elements in plant genomes. These findings provide a comprehensive overview of SNP distribution and establish a solid foundation for subsequent GWAS, facilitating more accurate identification and biological interpretation of candidate loci.

### 3.3 Identification of significant SNPs and candidate genes associated with Tartary buckwheat traits

GWAS was performed across five architectural traits in Tartary buckwheat, and revealed multiple significant genetic loci and their associated candidate genes. The significance threshold was set at P < 1×10^-5^, identifying a total of 890 significant SNP markers distributed across the genome (**Supplementary Table S2**) and mapping to 332 distinct genomic loci (**Supplementary Table S3**).

GWAS for PH were performed across four trait–environment combinations (**Figure 4**). Manhattan plots revealed that no significant associations surpassed the significance threshold (−log_10_*P* = 5) in the 24SM (PH_24SM) or 25SM (PH_25SM) environments, whereas prominent peaks were detected in the 25HZ environment (PH_25HZ) and in the multi-environment BLUE estimates (PH_BLUE). Quantile–quantile (Q–Q) plots showed that most trait–environment combinations had well-controlled type I error, with genomic inflation factors (λGC) close to 1.0 (range: 0.990–1.113; **Supplementary Table S4**). Notably, PH_25HZ (λGC = 1.234) and PH_BLUE (λGC = 1.115) exhibited modest upward deviation from the expected distribution. To investigate whether this inflation was attributable to population stratification, we performed PCA on all genotyped SNPs. The top 10 PC eigenvalues decreased gradually from 3.33 to 1.55, with a PC1/PC2 ratio of 1.31, indicating no dominant substructure among the accessions, which were all collected from the same geographic region. Therefore, the observed inflation in PH_25HZ and PH_BLUE is more likely attributable to the polygenic architecture of PH, a complex quantitative trait, combined with the increased statistical power of multi-environment BLUE estimates that integrate genetic signals across environments. The strongest associations were localized to an 88 kb region on Chr4 (lead SNP cluster Chr4: 31011474–31099420; minimum *P* = 2.19 × 10^-^¹¹, –log_10_*P* = 10.66) and to Chr4: 16478452 (*P* = 2.14 × 10^-^¹¹) (**Supplementary Table S5**). Within ±200 kb of lead SNPs, 21 high-confidence candidate genes were prioritized using a composite scoring scheme integrating GWAS significance, model-species homology, functional annotation, and cross-trait pleiotropy (**Table 4**). Notably, 15 of the 21 candidates (71.4%) exhibited cross-trait pleiotropy, with concurrent associations to NNMS or BNMS, indicating that a substantial fraction of PH-associated loci may represent shared genetic factors underlying multiple plant architectural traits rather than height-specific loci.

**Figure 4:**
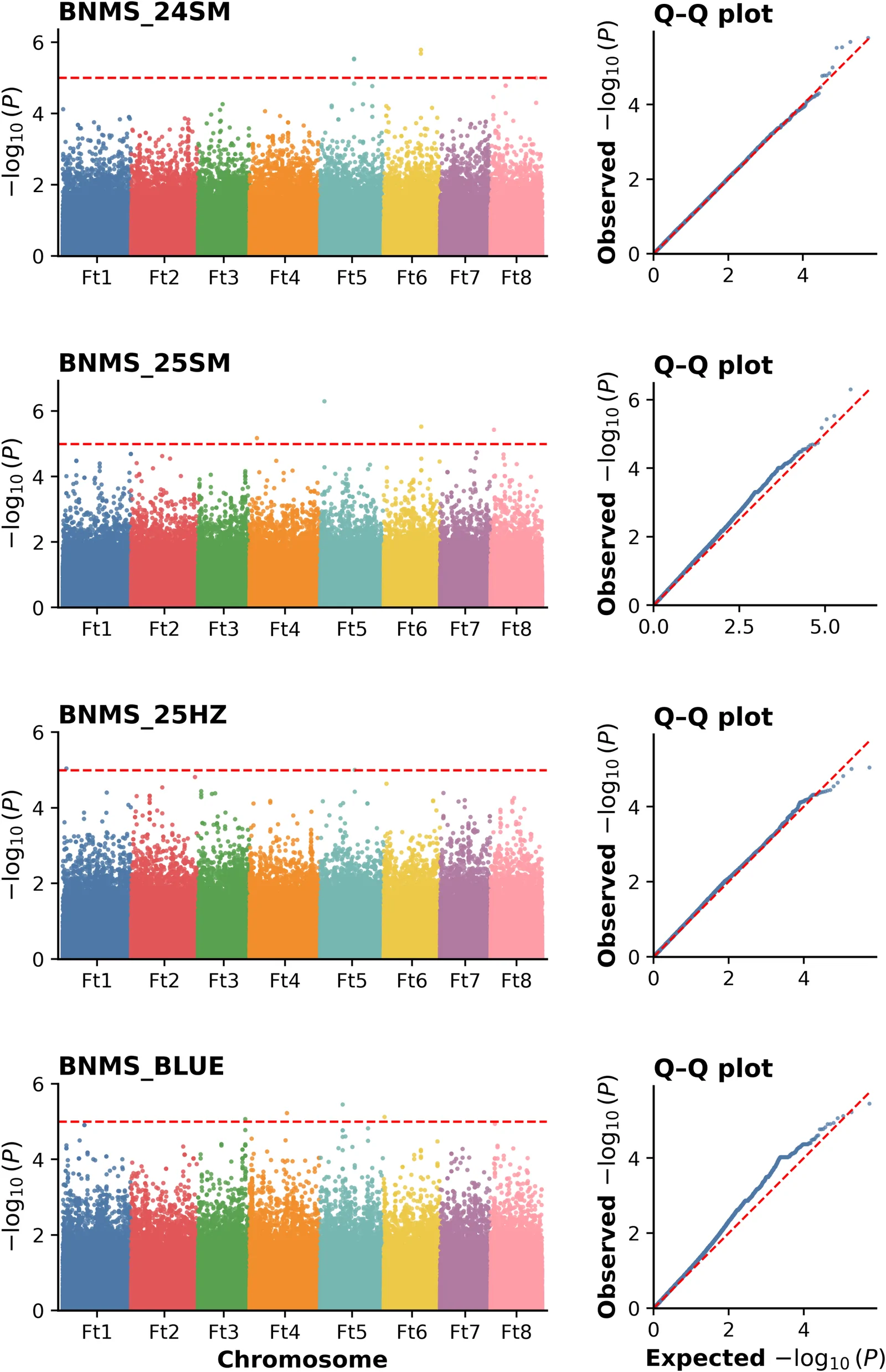
Genome-wide association analysis of PH in Tartary buckwheat. Manhattan plots showing −log_10_(*P*) values for each SNP across eight chromosomes (Chr1–Chr8). Right panels: corresponding quantile–quantile (Q–Q) plots. Rows from top to bottom represent PH measured at Songming 2024 (PH_24SM), Songming 2025 (PH_25SM), Huize 2025 (PH_25HZ), and multi-environment best linear unbiased estimates (PH_BLUE). The horizontal red dashed line indicates the significance threshold (−log_10_*P* = 5).

**Table 4:** Genomic positions and GWAS statistics of 21 prioritized candidate genes for PH in Tartary buckwheat.

Rank	Candidate gene ID	Chr	Lead SNP position (bp)	*P*-value	−log_10_*P*	Env.^a^	Pleio.^b^	CS^c^
1	FtPinG0007266400.01	4	31,099,420	2.19 × 10^-^¹¹	10.66	PH_25HZ; PH_BLUE	Y	0.955
2	FtPinG0004749000.01	4	31,099,420	2.19 × 10^-^¹¹	10.66	PH_25HZ	Y	0.947
3	FtPinG0008466000.01	4	16,478,452	2.14 × 10^-^¹¹	10.67	PH_25HZ	Y	0.922
4	FtPinG0004750100.01	4	31,099,420	2.19 × 10^-^¹¹	10.66	PH_25HZ	Y	0.922
5	FtPinG0008208800.01	4	35,079,438	5.46 × 10^-^¹¹	10.26	PH_25HZ; PH_BLUE	Y	0.922
6	FtPinG0005732300.01	5	6,260,249	7.79 × 10^-8^	7.11	PH_25HZ	Y	0.874
7	FtPinG0001787600.01	3	6,973,951	7.46 × 10^-9^	8.13	PH_25HZ	Y	0.872
8	FtPinG0006018700.01	1	35,745,631	1.35 × 10^-8^	7.87	PH_25HZ	N	0.854
9	FtPinG0009771900.01	2	18,090,159	1.24 × 10^-8^	7.91	PH_25HZ	Y	0.841
10	FtPinG0005361800.01	4	25,607,232	1.50 × 10^-8^	7.82	PH_25HZ	Y	0.838
11	FtPinG0009581800.01	4	30,494,228	3.40 × 10^-8^	7.47	PH_25HZ; PH_BLUE	Y	0.828
12	FtPinG0001007100.01	1	22,534,437	1.79 × 10^-9^	8.75	PH_25HZ	N	0.815
13	FtPinG0006605400.01	4	31,099,420	2.19 × 10^-^¹¹	10.66	PH_25HZ	Y	0.808
14	FtPinG0001545100.01	7	31,215,219	4.47 × 10^-7^	6.35	PH_25HZ	Y	0.804
15	FtPinG0004749800.01	4	31,099,420	2.19 × 10^-^¹¹	10.66	PH_25HZ	Y	0.802
16	FtPinG0005586300.01	8	34,345,763	4.46 × 10^-7^	6.35	PH_25HZ	Y	0.796
17	FtPinG0007761600.01	6	18,113,036	3.48 × 10^-6^	5.46	PH_25HZ	N	0.779
18	FtPinG0007088600.01	5	33,370,749	2.82 × 10^-6^	5.55	PH_BLUE	N	0.773
19	FtPinG0007198200.01	1	40,116,436	8.86 × 10^-8^	7.05	PH_25HZ	Y	0.766
20	FtPinG0001191100.01	8	46,470,129	5.20 × 10^-6^	5.28	PH_25HZ	N	0.765
21	FtPinG0005302200.01	4	11,146,192	5.79 × 10^-6^	5.24	PH_25HZ	N	0.764

^a^ Env., trait–environment combination(s) in which the association reached genome-wide significance: PH_25HZ = plant height measured at Huize 2025; PH_BLUE = plant height best linear unbiased estimate across environments. ^b^ Pleio., cross-trait pleiotropy (Y/N): the locus is also significantly associated with at least one other architectural trait (NNMS or BNMS). ^c^ CS, composite score integrating GWAS significance, model-species homology, functional annotation, and cross-trait pleiotropy.

The candidate genes encoded proteins with diverse predicted functions, including transcription factors (*TGA10*, *bZIP19*, *HAT14*, PLATZ-family transcription factor, and an SRM1-like homeodomain transcription factor), cell-wall-related proteins (*PGIP1* and a pectin lyase-like protein), transporters, protein kinases, ubiquitin-related proteins, and metabolic enzymes (**Table 5**). Five candidate genes, including *TGA10*, *PGIP1*, the pectin lyase-like gene homologous to Arabidopsis *AT3G57790*, an S-adenosyl-methionine-dependent methyltransferase, and *RABA4D*, were located within the same significant association region on Chr4 (Chr4: 31011474–31099420). Several of these genes may contribute to cell-wall modification, signaling regulation, or cell expansion-related processes.

**Table 5:** Functional annotation of the top 21 candidate genes for PH in Tartary buckwheat and their homologs in *Arabidopsis thaliana* and *Oryza sativa*.

Rank	Candidate gene ID	At homolog	At function	At id/cov (%)^a^	Os homolog	Os function	Os id/cov (%)^b^	Function class
1	FtPinG0007266400.01	AT5G06839 (*TGA10*)	bZIP transcription factor family protein	59.6/88	Os09g0489500 (*TGA2*)	bZIP transcription factor TGA2	51.0/85	bZIP TF
2	FtPinG0004749000.01	AT5G06860 (*PGIP1*)	Polygalacturonase-inhibiting protein 1	51.3/100	Os05g0104700 (*PGIP1*)	Polygalacturonase inhibitor 1	43.2/98	LRR protein
3	FtPinG0008466000.01	AT4G35040 (*bZIP19*)	Basic leucine zipper transcription factor 19	61.6/85	Os06g0716800 (*bZIP19*)	Basic leucine zipper transcription factor 19	56.0/92	bZIP TF
4	FtPinG0004750100.01	AT3G57790	Pectin lyase-like superfamily protein	67.5/95	Os09g0487600 (*PG*)	Probable polygalacturonase	65.1/90	Cell wall (pectin)
5	FtPinG0008208800.01	AT1G52310	Protein kinase containing a C-type lectin domain	62.8/91	Os01g0104000 (*C-typ*e lectin *RLK*)	C-type lectin receptor-like protein kinase	63.2/91	Kinase
6	FtPinG0005732300.01	AT5G08520	Duplicated homeodomain-like superfamily protein	53.7/67	Os04g0676700 (*SRM1*)	Homeodomain-like transcription factor SRM1	52.5/67	Homeodomain TF
7	FtPinG0001787600.01	AT3G12280 (*RBR1*)	Retinoblastoma-related protein 1	67.0/96	Os08g0538700 (*RBR1*)	Retinoblastoma-related protein 1	59.5/98	Cell cycle
8	FtPinG0006018700.01	AT2G37840	Protein kinase superfamily protein	58.6/99	Os03g0268200 (*ATG1c*)	Serine/threonine-protein kinase ATG1c	47.9/95	Kinase
9	FtPinG0009771900.01	AT1G34580	Major facilitator superfamily protein	51.0/95	Os07g0106200 (*MST3*)	Sugar transporter protein MST3	46.8/93	Transporter
10	FtPinG0005361800.01	AT5G42190	SCF E3 ubiquitin ligase subunit SKP1/ASK1-like protein	39.0/70	Os09g0539500 (*SKP1*-like *1*)	SKP1-like protein 1, a subunit of the SCF E3 ubiquitin ligase complex	36.2/67	Ubiquitin ligase
11	FtPinG0009581800.01	AT5G06710 (*HAT14*)	Homeobox-leucine zipper protein *HAT14*	50.7/98	Os04g0548700 (*HOX27*)	Homeobox-leucine zipper protein HOX27	75.0/52	HD-ZIP TF
12	FtPinG0001007100.01	AT1G31040	PLATZ transcription factor family protein	45.0/74	Os06g0666100 (*LOC4341780*)	Uncharacterized protein with predicted PLATZ transcription factor-related function	39.2/87	PLATZ TF
13	FtPinG0006605400.01	AT2G31740	SAM-dependent methyltransferase superfamily protein	57.4/99	Os04g0379300 (*MTL13*)	Methyltransferase-like protein 13	55.1/96	Methyltransferase
14	FtPinG0001545100.01	AT2G37010 (*NAP12*)	Non-intrinsic ABC protein 12	55.5/100	Os11g0416900 (*ABCG28*)	ABC transporter G family member 28	53.7/99	ABC transporter
15	FtPinG0004749800.01	AT3G12160 (*RABA4D*)	RAB GTPase homolog A4D	78.6/98	Os10g0377400 (*RGP1*)	Ras-related GTP-binding protein RGP1	73.2/99	GTPase
16	FtPinG0005586300.01	AT3G47420 (*G3Pp1*)	Glycerol-3-phosphate transporter 1	65.1/100	Os04g0555300 (*G3P* transporter *1*)	Putative glycerol-3-phosphate transporter 1	62.1/98	Transporter
17	FtPinG0007761600.01	AT3G59420 (*CR4*)	Crinkly4 receptor-like kinase	66.2/99	Os03g0637800 (*CR4*)	Serine/threonine-protein kinase-like protein CR4	65.5/99	Kinase
18	FtPinG0007088600.01	AT3G28345 (*ABCB15*)	ATP-binding cassette transporter B15	62.9/99	Os02g0190300 (*MDR* protein)	Putative multidrug-resistance ABC transporter	59.4/99	ABC transporter
19	FtPinG0007198200.01	AT4G35520 (*MLH3*)	MutL protein homolog 3	59.4/90	Os09g0551900 (*MLH3*)	DNA mismatch-repair protein MLH3	53.4/91	ATPase/DNA repair
20	FtPinG0001191100.01	AT5G46330 (*FLS2*)	Leucine-rich repeat receptor-like protein kinase FLS2	40.6/96	Os04g0618700 (*FLS2*)	Leucine-rich repeat receptor-like serine/threonine-protein kinase FLS2	43.5/97	LRR-RLK
21	FtPinG0005302200.01	AT2G45550 (*CYP76C4*)	Cytochrome P450 family 76 subfamily C polypeptide 4	55.9/98	Os03g0248200 (*geraniol 8-hydroxylase*)	Geraniol 8-hydroxylase, a cytochrome P450 monooxygenase	46.9/98	P450

^a^At id/cov (%), percentage amino acid sequence identity and query coverage between the candidate protein and its best-matching Arabidopsis thaliana homolog. ^b^Os id/cov (%), percentage amino acid sequence identity and query coverage between the candidate protein and its best-matching Oryza sativa homolog. At, Arabidopsis thaliana; Os, Oryza sativa; TF, transcription factor; bZIP, basic leucine zipper; LRR, leucine-rich repeat; RLK, receptor-like kinase; HD-ZIP, homeodomain-leucine zipper; PLATZ, plant AT-rich sequence- and zinc-binding protein; ABC, ATP-binding cassette; SCF, SKP1–Cullin–F-box; P450, cytochrome P450.

GWAS for NNMS were performed across four trait–environment combinations (**Figure 5**). Only sporadic marginal signals were observed in Songming 2025 (NNMS_25SM) and Songming 2024 (NNMS_24SM). In contrast, prominent and highly significant peaks were detected in the Huize 2025 environment (NNMS_25HZ) and in the multi-environment BLUE estimates (NNMS_BLUE), with the strongest signals concentrated on chromosome 4 (−log_10_*P* > 10). Q–Q plots indicated well-controlled inflation for most trait–environment combinations (λGC range: 0.990–1.113). However, NNMS_25HZ λGC=1.266 and NNMS_BLUE λGC=1.107 showed notable upward deviation from the expected null distribution. As discussed above, PCA of all genotyped SNPs revealed no dominant population substructure among the accessions (PC1/PC2 eigenvalue ratio = 1.31), suggesting that population stratification is unlikely to be the primary driver of this inflation. Instead, the elevated λGC values for NNMS_25HZ and NNMS_BLUE are consistent with the highly polygenic nature of node number at main stem, a complex architectural trait, and the enhanced statistical power of multi-environment BLUE estimates. The concentration of the strongest signals on chromosome 4 (−log_10_*P* > 10) further supports the presence of genuine genetic associations rather than systematic technical artifact. Significant signals were distributed across chromosomes 1, 2, 3, 4, 5, 6, 7, and 8 (**Supplementary Table S6**). The two strongest associations were both localized to Chr4: an 88-kb significant association region at chromosome 4: 31011474–31099420 (minimum *P* = 2.19 × 10^-^¹¹, −log_10_*P* = 10.66) and a single SNP at Chr4: 16478452 (*P* = 2.14 × 10^-^¹¹, −log_10_*P* = 10.67). Within ±200 kb of all lead SNPs, 113 candidate genes were prioritized by the composite scoring scheme, of which 22 were classified as Tier 1 candidates with strong GWAS support and high-confidence homology to characterized model-species genes (**Tables 6**, **7**). Strikingly, 10 of the 22 Tier 1 candidates (45.5%) were localized to chromosome chromosome 4, substantially exceeding the genome-wide average of 1.7 Tier 1 candidates per chromosome and confirming Chr4 as a major regulatory hotspot for node-number variation. Of the 22 Tier 1 candidates, 15 (68.2%) also showed significant associations with PH and/or BNMS, indicating extensive genetic coupling between NNMS and overall plant architecture, while seven NNMS-specific candidates pointed to regulatory layers—most notably gibberellin and ethylene signaling—that appear to act preferentially on node initiation.

**Figure 5:**
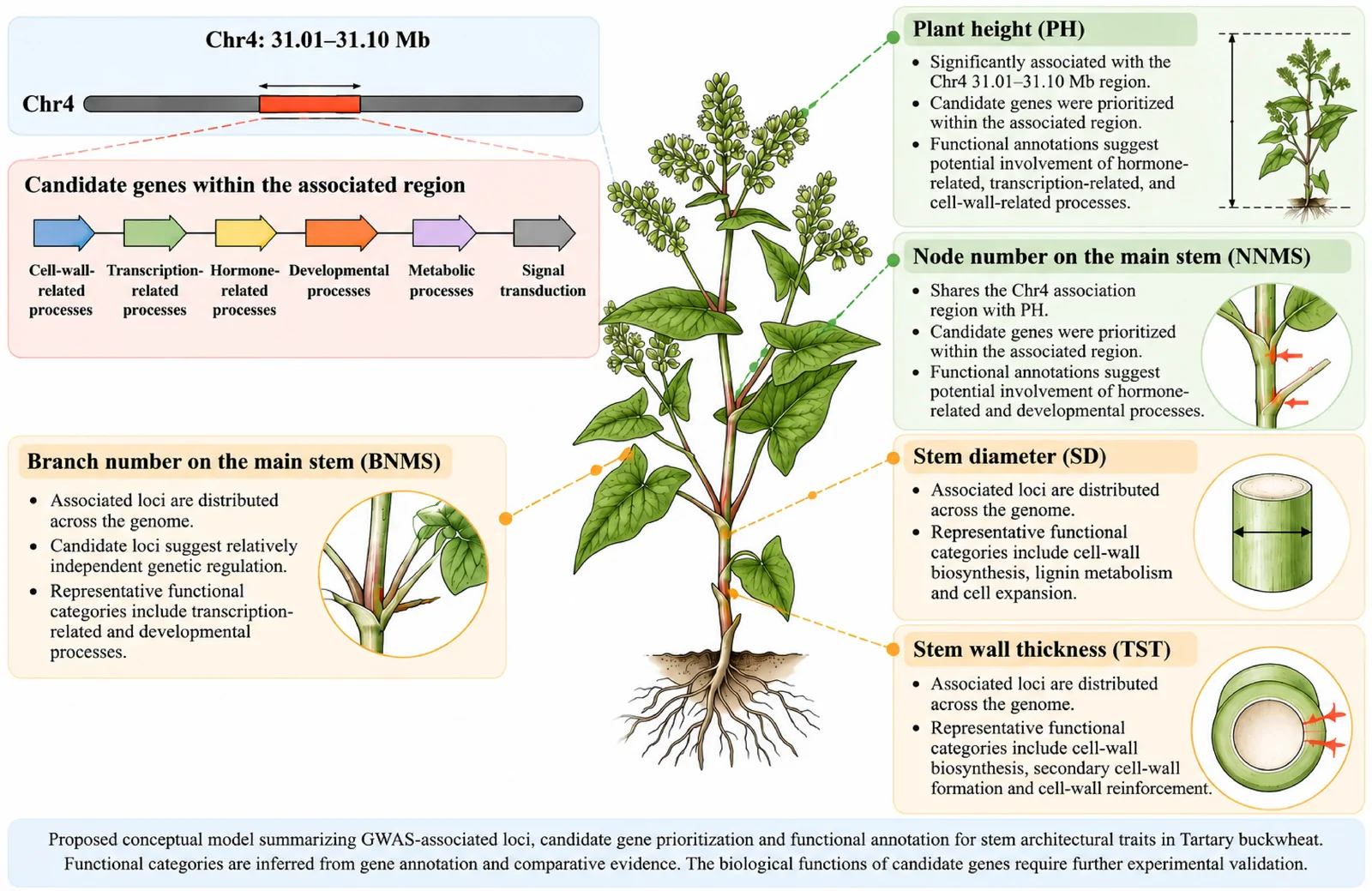
Manhattan plots and quantile–quantile (Q–Q) plots for genome-wide association analysis of NNMS in Tartary buckwheat. Left panels: Manhattan plots showing −log_10_(*P*) for each SNP across eight chromosomes (Chr1–Chr8). The horizontal red dashed line indicates the significance threshold (−log_10_*P* = 5). Right panels: corresponding Q–Q plots comparing observed (y-axis) versus expected (x-axis) −log_10_*P* values under the null hypothesis of no association; the red dashed diagonal line represents the expected uniform distribution.

**Table 6:** Genomic positions and GWAS statistics of 22 Tier 1 candidate genes for NNMS in Tartary buckwheat.

Rank	Candidate gene ID	Chr	Lead SNP position (bp)	*P-value*	−log_10_*P*	Env.^a^	Pleio.^b^	CS^c^
1	FtPinG0007266400.01	4	31,099,420	2.19 × 10^-^¹¹	10.66	NNMS_25HZ; NNMS_BLUE	Y	0.955
2	FtPinG0004749000.01	4	31,099,420	2.19 × 10^-^¹¹	10.66	NNMS_25HZ; NNMS_BLUE	Y	0.947
3	FtPinG0008466000.01	4	16,478,452	2.14 × 10^-^¹¹	10.67	NNMS_25HZ; NNMS_BLUE	Y	0.922
4	FtPinG0004750100.01	4	31,099,420	2.19 × 10^-^¹¹	10.66	NNMS_25HZ; NNMS_BLUE	Y	0.922
5	FtPinG0008208800.01	4	35,079,438	5.46 × 10^-^¹¹	10.26	NNMS_25HZ; NNMS_BLUE	Y	0.922
6	FtPinG0005732300.01	5	6,260,249	7.79 × 10^-8^	7.11	NNMS_25HZ	Y	0.874
7	FtPinG0001787600.01	3	6,973,951	7.46 × 10^-9^	8.13	NNMS_25HZ; NNMS_BLUE	Y	0.872
8	FtPinG0004749900.01	4	31,011,474	2.68 × 10^-9^	8.57	NNMS_BLUE	N	0.86
9	FtPinG0009771900.01	2	18,090,159	1.24 × 10^-8^	7.91	NNMS_25HZ; NNMS_BLUE	Y	0.841
10	FtPinG0005361800.01	4	25,607,232	1.50 × 10^-8^	7.82	NNMS_25HZ; NNMS_BLUE	Y	0.838
11	FtPinG0009581800.01	4	30,494,228	3.40 × 10^-8^	7.47	NNMS_25HZ; NNMS_BLUE	Y	0.828
12	FtPinG0001433100.01	6	8,657,347	2.66 × 10^-7^	6.57	NNMS_25HZ	N	0.825
13	FtPinG0004159800.01	8	44,757,002	3.20 × 10^-7^	6.49	NNMS_25HZ; NNMS_BLUE	N	0.814
14	FtPinG0006605400.01	4	31,099,420	2.19 × 10^-^¹¹	10.66	NNMS_25HZ; NNMS_BLUE	Y	0.808
15	FtPinG0001545100.01	7	31,215,219	4.47 × 10^-7^	6.35	NNMS_25HZ	Y	0.804
16	FtPinG0004749800.01	4	31,099,420	2.19 × 10^-^¹¹	10.66	NNMS_25HZ; NNMS_BLUE	Y	0.802
17	FtPinG0005586300.01	8	34,345,763	4.46 × 10^-7^	6.35	NNMS_25HZ	Y	0.796
18	FtPinG0006401900.01	1	38,014,159	9.24 × 10^-7^	6.03	NNMS_25HZ	N	0.787
19	FtPinG0002120100.01	3	55,003,072	2.09 × 10^-6^	5.68	NNMS_BLUE	N	0.777
20	FtPinG0008108900.01	1	42,160,313	2.34 × 10^-6^	5.63	NNMS_25HZ	N	0.775
21	FtPinG0005324100.01	7	32,825,255	3.08 × 10^-6^	5.51	NNMS_25HZ	N	0.772
22	FtPinG0007198200.01	1	40,116,436	8.86 × 10^-8^	7.05	NNMS_25HZ; NNMS_BLUE	Y	0.766

^a^Env., trait–environment combination(s) reaching genome-wide significance: NNMS_25HZ = node number on the main stem measured at Huize 2025; NNMS_BLUE = multi-environment BLUE estimate. ^b^Pleio., cross-trait pleiotropy (Y/N): the locus is also significantly associated with PH and/or BNMS. ^c^CS, composite score integrating GWAS significance, model-species homology, functional annotation, and cross-trait pleiotropy.

**Table 7:** Functional annotation of the top 22 candidate genes for NNMS in Tartary buckwheat and their homologs in *Arabidopsis thaliana* and *Oryza sativa*.

Rank	Candidate gene ID	At homolog	At function	At id/cov (%)^a^	Os homolog	Os function	Os id/cov (%)^b^	Function class
1	FtPinG0007266400.01	AT5G06839 (*TGA10*)	bZIP transcription factor family protein	59.6/88	Os09g0489500 (*TGA2*)	bZIP transcription factor	51.0/85	bZIP TF
2	FtPinG0004749000.01	AT5G06860 (*PGIP1*)	Polygalacturonase inhibiting protein 1	51.3/100	Os05g0104700 (*PGIP1*)	Polygalacturonase inhibitor 1	43.2/98	LRR-RLK
3	FtPinG0008466000.01	AT4G35040 (*bZIP19*)	Basic leucine zipper transcription factor	61.6/85	Os06g0716800 (*bZIP19*)	Basic leucine zipper transcription factor 19	56.0/92	bZIP TF
4	FtPinG0004750100.01	AT3G57790	Pectin lyase-like superfamily protein	67.5/95	Os09g0487600	Probable polygalacturonase	65.1/90	Cell wall (pectin)
5	FtPinG0008208800.01	AT1G52310	Protein kinase/C-type lectin domain protein	62.8/91	Os01g0104000	C-type lectin receptor-like protein kinase	63.2/91	Kinase
6	FtPinG0005732300.01	AT5G08520	Duplicated homeodomain-like superfamily protein	53.7/67	Os04g0676700 (*SRM1*)	Transcription factor SRM1	52.5/67	HD-ZIP/MYB TF
7	FtPinG0001787600.01	AT3G12280 (RBR1)	Retinoblastoma-related protein 1	67.0/96	Os08g0538700 (*RBR1*)	Retinoblastoma-related protein 1	59.5/98	Cell cycle
8	FtPinG0004749900.01	AT3G57800 (*bHLH60*)	Basic helix-loop-helix DNA-binding superfamily protein	50.9/99	Os06g0613500 (*bHLH48*)	Basic helix-loop-helix transcription factor	57.3/50	bHLH TF
9	FtPinG0009771900.01	AT1G34580	Major facilitator superfamily protein	51.0/95	Os07g0106200 (*MST3*)	Sugar transporter MST3	46.8/93	Transporter
10	FtPinG0005361800.01	AT5G42190	SCF E3 ubiquitin ligase subunit SKP1/ASK1-like	39.0/70	Os09g0539500	SKP1-like protein 1	36.2/67	Ubiquitin ligase
11	FtPinG0009581800.01	AT5G06710 (*HAT14*)	Homeobox-leucine zipper protein	50.7/98	Os04g0548700 (*HOX27*)	Homeobox-leucine zipper protein	75.0/52	HD-ZIP TF
12	FtPinG0001433100.01	AT2G39510 (*UMAMIT14*)	Nodulin MtN21/EamA-like transporter family protein	60.8/97	Os10g0210500	WAT1-related transporter	49.7/99	Transporter
13	FtPinG0004159800.01	AT3G24550 (*PERK1*)	Proline extensin-like receptor kinase 1	70.3/81	Os05g0218400 (*PERK1*)	Proline-rich receptor-like protein kinase PERK1	70.7/73	Kinase
14	FtPinG0006605400.01	AT2G31740	SAM-dependent methyltransferase superfamily protein	57.4/99	Os04g0379300	Methyltransferase-like protein 13	55.1/96	Methyltransferase
15	FtPinG0001545100.01	AT2G37010 (*NAP12*)	Non-intrinsic ABC protein 12	55.5/100	Os11g0416900 (*ABCG28*)	ABC transporter G family member 28	53.7/99	ABC transporter
16	FtPinG0004749800.01	AT3G12160 (*RABA4D*)	RAB GTPase homolog A4D	78.6/98	Os10g0377400 (*RGP1*)	Ras-related GTP-binding protein	73.2/99	GTPase
17	FtPinG0005586300.01	AT3G47420 (*G3Pp1*)	Glycerol-3-phosphate transporter 1	65.1/100	Os04g0555300	Glycerol-3-phosphate transporter	62.1/98	Transporter
18	FtPinG0006401900.01	AT5G03730 (*CTR1*)	Protein kinase involved in ethylene signaling	66.7/99	Os09g0566550 (*CTR1*)	Serine/threonine protein kinase CTR1	62.0/99	ET signal/Kinase
19	FtPinG0002120100.01	AT4G23180 (*CRK10*)	Cysteine-rich receptor-like protein kinase 10	53.1/99	Os07g0537000 (*CRK6*)	Cysteine-rich receptor-like protein kinase 6	46.0/100	Kinase
20	FtPinG0008108900.01	AT3G11440 (*MYB65*)	Myb domain protein 65	35.4/95	Os01g0812000 (*GAMYB*)	GAMYB transcription factor	39.1/89	MYB TF
21	FtPinG0005324100.01	AT1G73670 (*MPK15*)	Mitogen-activated protein kinase 15	78.3/99	Os05g0576800 (*MPK17*)	Mitogen-activated protein kinase 17	79.4/99	Kinase
22	FtPinG0007198200.01	AT4G35520 (*MLH3*)	MutL protein homolog 3	59.4/90	Os09g0551900 (*MLH3*)	DNA mismatch repair protein MLH3	53.4/91	ATPase/DNA repair

^a^At id/cov (%), percentage amino acid sequence identity and query coverage between the candidate protein and its best-matching *Arabidopsis thaliana* homolog. ^b^Os id/cov (%), percentage amino acid sequence identity and query coverage between the candidate protein and its best-matching *Oryza sativa* homolog. At, *Arabidopsis thaliana*; Os, *Oryza sativa*; TF, transcription factor; RLK, receptor-like kinase; LRR-RLK, leucine-rich repeat receptor-like kinase; HD-ZIP, homeodomain-leucine zipper; MYB, myeloblastosis transcription factor; bZIP, basic leucine zipper; bHLH, basic helix-loop-helix; ABC transporter, ATP-binding cassette transporter; ET, ethylene; ATPase, adenosine triphosphatase.

Genome-wide association analyses for BNMS were performed across four trait–environment combinations (**Figure 6**). Unlike PH and NNMS, which showed concentrated and highly significant signals predominantly in a single environment, BNMS associations were dispersed across multiple environments with modest signal magnitude. Significant SNPs exceeding the significance threshold (−log_10_*P* ≥ 5) were detected in BNMS_24SM, BNMS_25SM, BNMS_25HZ, and BNMS_BLUE, distributed across chromosomes 1, 3, 4, 5, 6, and 8. Q–Q plots confirmed minimal genomic inflation across all four analyses, consistent with a more polygenic genetic architecture for branching compared with PH and NNMS. Within ±200 kb of lead SNPs, three Tier 1 candidate genes were prioritized by the composite scoring scheme (**Tables 8**, **9**). These encoded an ABC transporter (*ABCB15* orthologue), a cytochrome P450 involved in phenylpropanoid metabolism (*CYP98A3*), and an AP2/ERF transcription factor (*TDR1*). The complete set of BNMS candidates across all priority tiers is provided in **Supplementary Table S7**.

**Figure 6:**
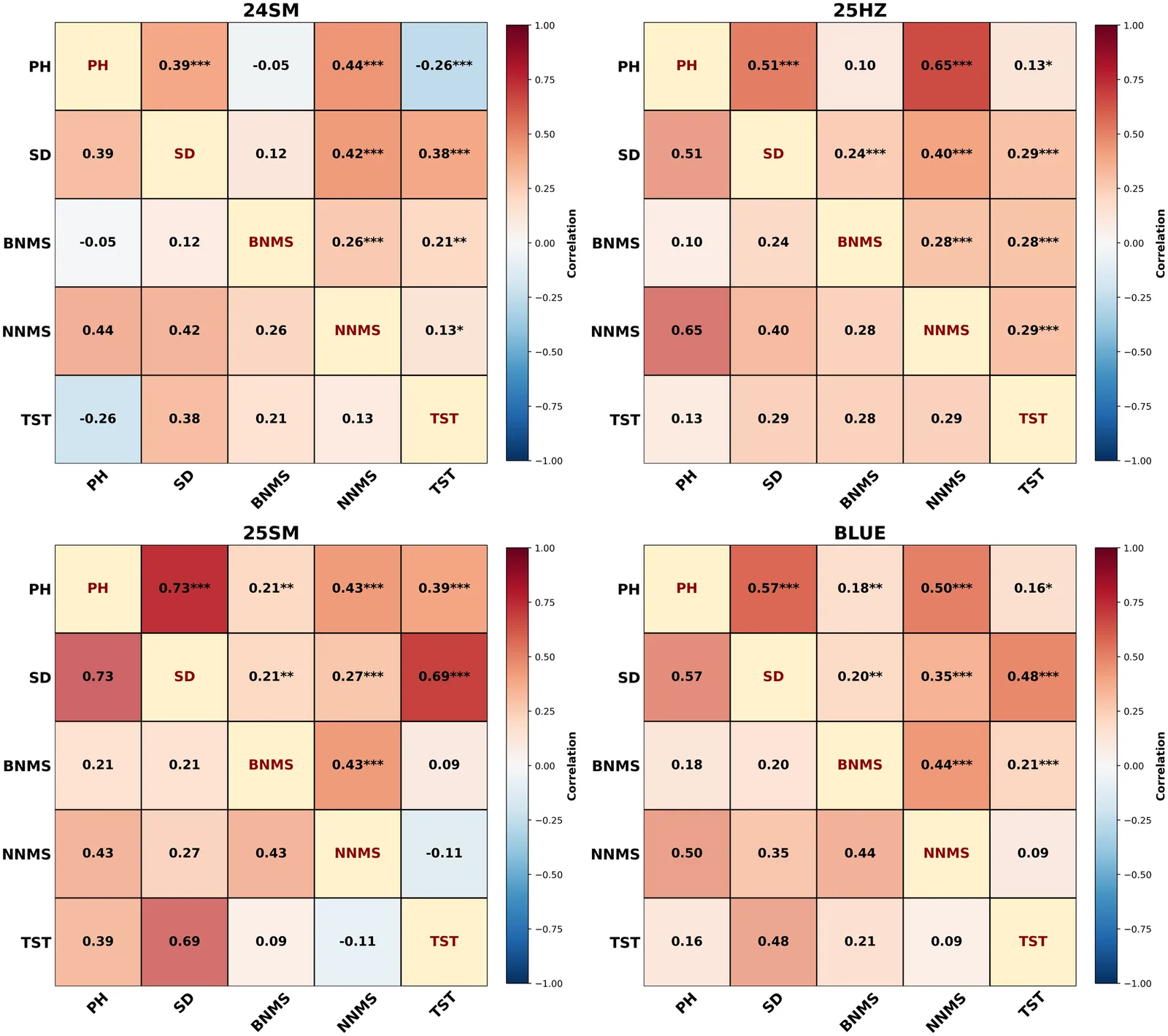
Manhattan plots and quantile–quantile (Q–Q) plots for genome-wide association analysis of BNMS in Tartary buckwheat. Manhattan plots showing −log_10_(*P*) for each SNP plotted against genomic position across eight chromosomes (Chr1–Chr8). The horizontal red dashed line indicates the significance threshold (−log_10_*P* = 5). Right panels: corresponding Q–Q plots comparing observed (y-axis) versus expected (x-axis) −log_10_(*P*) values under the null hypothesis of no association; the red dashed diagonal line represents the expected uniform distribution.

**Table 8:** Genomic positions and GWAS statistics of candidate genes for BNMS in Tartary buckwheat.

Rank	Candidate gene ID	Chr	Lead SNP position (bp)	*P-value*	−log_10_*P*	Env.^a^	Pleio.^b^	CS^c^
1	FtPinG0007853900.01	5	33,408,868	2.93 × 10^-6^	5.53	BNMS_24SM	N	0.781
2	FtPinG0002981600.01	6	34,836,326	2.99 × 10^-6^	5.52	BNMS_25SM	N	0.772
3	FtPinG0004938100.01	4	28,150,886	6.00 × 10^-6^	5.22	BNMS_BLUE	N	0.764

^a^Env., trait–environment combination in which the association reached genome-wide significance: BNMS_24SM = Songming 2024; BNMS_25SM = Songming 2025; BNMS_BLUE = multi-environment BLUE estimate. ^b^Pleio., cross-trait pleiotropy (Y/N): the locus is also significantly associated with PH and/or NNMS. ^c^CS, composite score integrating GWAS significance, model-species homology, functional annotation, and cross-trait pleiotropy.

**Table 9:** Functional annotation of the top 3 candidate genes for BNMS in Tartary buckwheat and their homologs in *Arabidopsis thaliana* and *Oryza sativa*.

Rank	Candidate gene ID	At homolog	At function	At id/cov (%)^a^	Os homolog	Os function	Os id/cov (%)^b^	Function class
1	FtPinG0007853900.01	AT3G28345 (*ABCB15*)	ABC transporter family protein	64.2/99	Os02g0190300	Putative multidrug resistance protein	60.5/99	ABC transporter
2	FtPinG0002981600.01	AT2G40890 (*CYP98A3*)	Cytochrome P450, family 98, subfamily A, polypeptide 3	72.1/96	Os05g0494000 (*CYP98A1*)	Cytochrome P450 98A1	65.8/96	P450
3	FtPinG0004938100.01	AT3G23230 (*TDR1*)	Integrase-type DNA-binding superfamily protein (AP2/ERF)	59.7/95	Os03g0150200 (*ERF096*)	Ethylene-responsive transcription factor ERF096	55.6/87	AP2/ERF TF

^a^At id/cov (%), amino acid sequence identity (%)/query coverage (%) between the tartary buckwheat candidate protein and its best-matched A. thaliana homolog. ^b^Os id/cov (%), amino acid sequence identity (%)/query coverage (%) between the tartary buckwheat candidate protein and its best-matched O. sativa homolog. ABC, ATP-binding cassette; P450, cytochrome P450 monooxygenase; AP2/ERF, APETALA2/ethylene-responsive factor; TF, transcription factor.

For SD, only six candidate genes were prioritized within ±200 kb of significant SNPs across the four environments, with the strongest association at Chr1: 66713654 in SD_25HZ (*P* = 3.32 × 10^-7^, −log_10_*P* = 6.48); for TST, six candidates were likewise identified, with the strongest signal at Chr8: 16564004 in TST_BLUE (*P* = 1.30 × 10^-8^, −log_10_*P* = 7.89). No candidate for either trait reached the Tier 1 priority threshold, and signals were dispersed across multiple chromosomes without evidence of clustering, consistent with a highly polygenic genetic architecture for both stem-mechanical traits. Despite the overall modest signal strength, two candidates provided mechanistically informative leads. For SD, the highest composite-ranked gene was FtPinG0008501600 on Chr4 (composite score = 0.70, Tier 2), identified as the orthologue of *FBH4* (*AT2G42280*), a bHLH transcription factor implicated in photoperiodic and growth-related transcriptional regulation. For TST, the top candidate was FtPinG0008957400 on Chr5 (composite score = 0.76, Tier 2), an orthologue of an *Arabidopsis thaliana* pectin-methylesterase inhibitor (*PMEI*; *AT2G01610*; identity = 54.1%, coverage = 84%). PMEs and their cognate inhibitors constitute a central regulatory switch governing pectin methyl-esterification status in the primary cell wall: variation at this locus could plausibly translate into differences in Ca²^+^-mediated cross-linking, cell-wall stiffness, and ultimately stem-wall thickness during secondary growth. The complete candidate lists for SD and TST, including GWAS statistics, homology evidence, and functional annotations, are provided in **Supplementary Tables S8**, **S9**.

Because the five traits were analyzed in the same panel with the same SNP set, we compared the loci and candidate genes recovered for each trait. Shared genetic control between PH and NNMS was extensive: fifteen genes were common to the two candidate sets, equivalent to 71.4% of the PH candidates and 68.2% of the Tier 1 NNMS candidates, and included the five genes located at the Chr4: 31,099,420 lead SNP together with FtPinG0008466000 at Chr4: 16,478,452 and FtPinG0008208800 at Chr4: 35,079,438. Nine of these fifteen shared genes (60.0%) lie on Chr4, consistent with the very strong phenotypic and genetic correlation between PH and NNMS. Beyond the PH–NNMS module, an ABCB15-type ABC transporter was independently implicated in PH (FtPinG0007088600) and BNMS (FtPinG0007853900), although through different paralogues, and FtPinG0007198200 (MLH3) was associated with both PH and NNMS. In contrast, no Tier 1 candidate for BNMS, SD or TST coincided with the Chr4 block, and SD and TST shared no candidate gene with PH or NNMS.

The GWAS results for the five architectural traits revealed a series of associated loci, candidate genes, and functional annotations, which together outline the genetic basis of plant architecture regulation in Tartary buckwheat (**Figure 7**). The identified loci exhibited both pleiotropic and trait-specific effects, with candidate genes falling into several functional categories, including cell wall-related processes, transcriptional regulation, hormone-related pathways, developmental processes, metabolism, and signal transduction. Collectively, these findings indicate that plant architecture is governed by a coordinated network of genetic pathways encompassing growth, development, branching, and stem strength. Pyramiding favorable alleles across these pathways may therefore offer promising breeding targets for enhancing lodging resistance and yield potential.

**Figure 7:**
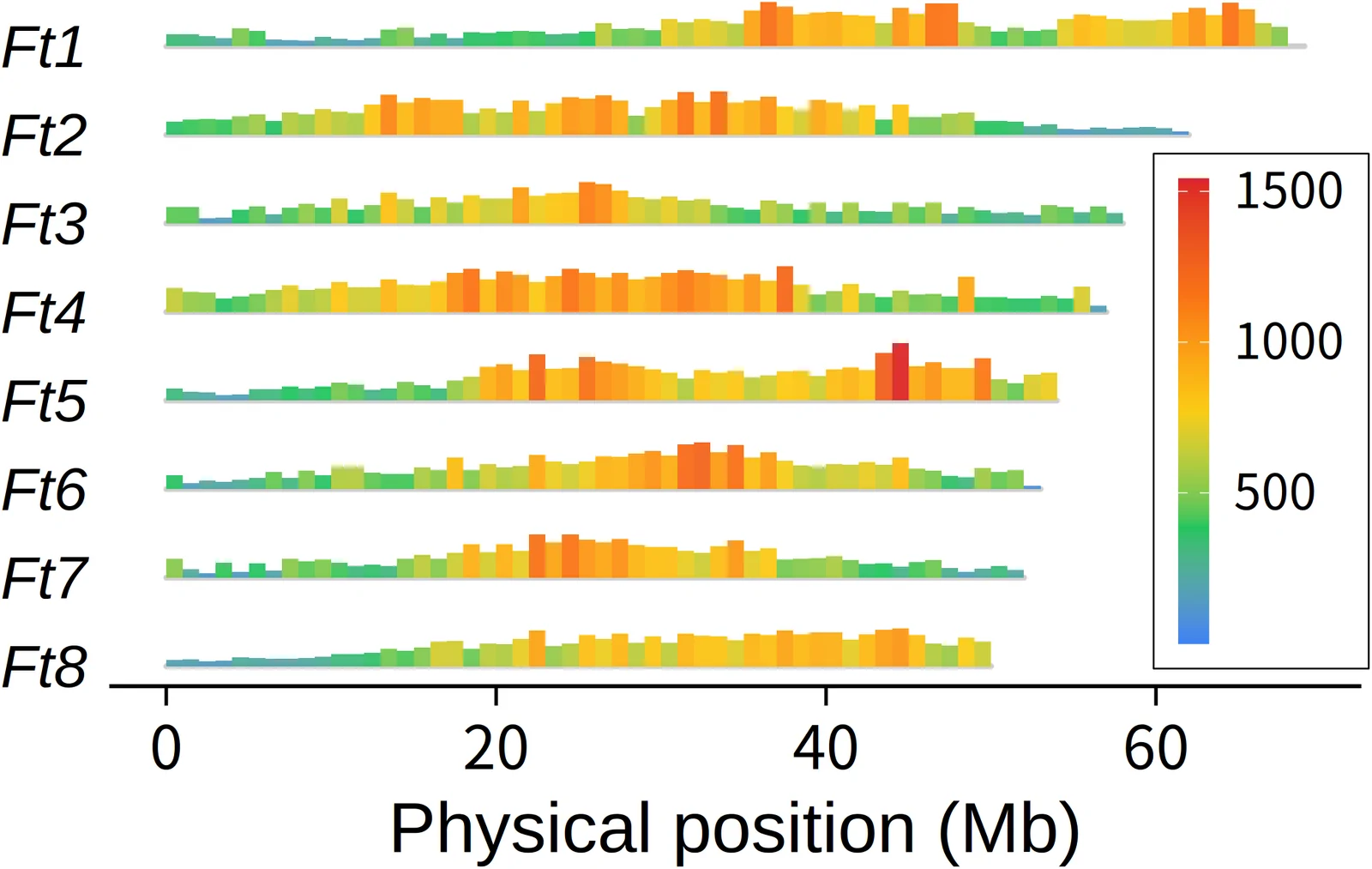
Proposed conceptual model summarizing GWAS-associated loci, candidate gene prioritization and functional annotation for stem architectural traits in Tartary buckwheat.

## 4 Discussion

Our study revealed broad variation in all five stem architecture traits among the 244 Tartary buckwheat accessions. PH and NNMS had moderate coefficients of variation, whereas BNMS showed relatively higher variation. This level of phenotypic diversity provides a useful basis for GWAS (Tibbs Cortes et al., 2021). The nearly normal distributions of these traits also suggest quantitative inheritance and possible environmental influence, making them suitable for mixed-model association analysis (Tam et al., 2019). The consistent positive correlation between PH and NNMS indicates that PH in Tartary buckwheat is closely related to node production. Because PH depends on both node number and internode length, accessions with more nodes on the main stem generally tended to be taller. The overlap of several GWAS signals between PH and NNMS further supports this relationship. The moderate positive correlation between PH and SD suggests that taller plants may also develop thicker stems, possibly as a structural adjustment to support increased PH and reduce lodging risk (Kashiwagi et al., 2008; Qin et al., 2017).

By contrast, TST was only weakly correlated with the other traits, suggesting that stem wall thickness may be controlled partly by processes related to cell-wall deposition and lignification rather than by the same factors that determine overall stem size (Zhang et al., 2021b). BNMS was almost independent of the PH–NNMS relationship, suggesting that branch formation may involve different regulatory mechanisms from those controlling NNMS and elongation. This agrees with previous findings that axillary bud growth is regulated mainly by auxin, strigolactone, and cytokinin signaling, whereas internode elongation is more closely related to the gibberellin pathway (Wang et al., 2018; Wang and Wang, 2022).

In this study, a major genomic region on Chr4 was consistently associated with both PH and NNMS, suggesting that this region may play an important role in regulating stem development in Tartary buckwheat. The co-localization of association signals and the high proportion of shared loci between these two traits indicate that PH and NNMS may be at least partly controlled by common genetic factors. Within the Chr4 interval, several candidate genes are involved in cell-wall modification and related processes, including *PGIP1* and a pectin lyase-like gene. Pectin modification has been widely reported to influence cell-wall extensibility and cell expansion by altering homogalacturonan methylesterification and Ca²^+^-mediated cross-linking (Wormit and Usadel, 2018; Wang et al., 2025a). In addition, pectin dynamics have been linked to organ initiation and boundary formation in the shoot apical meristem (Zhang et al., 2025). These studies suggest that variation in pectin-related genes could affect both internode elongation and node formation, providing a possible explanation for the shared genetic signals observed for PH and NNMS.

In addition to cell-wall-related genes, *RABA4D*, which is involved in vesicle trafficking, was also identified in this region. Vesicle transport plays an essential role in delivering cell-wall components and remodeling enzymes during growth (Kirchhelle et al., 2016) and has been implicated in polarized cell expansion. The identification of multiple transcription factors, including *TGA10*, *bZIP19*, *HAT14*, *MYB65*, and *bHLH48*, further supports the biological relevance of our GWAS results. Although their specific functions have not yet been experimentally validated in Tartary buckwheat, homologous genes in *Arabidopsis* and rice participate in transcriptional regulation of hormone responses, meristem activity, and organ growth. Their co-localization with highly significant GWAS signals therefore strengthens the hypothesis that they contribute to the regulation of plant architecture in Tartary buckwheat.

A comparison with cereals suggests that part of this machinery is functionally conserved across angiosperms. The sorghum GWAS outlined in the Introduction identified 321 significant SNPs, 22 pleiotropic loci and three hotspots on chromosomes 6, 7 and 9 that, together with Dw1, Dw2 and Dw3, control PH, SD and stem volume (Singh et al., 2025). Several sorghum candidates are functionally convergent with those identified here: the season-stable pleiotropic locus S03_15463061 lies next to a pectinesterase and an endoglucanase, and a tiller-number locus on chromosome 10 flanks an invertase/pectin methylesterase, indicating that pectin modification and cell-wall remodeling are recurrent determinants of stem architecture in grasses as they appear to be in Tartary buckwheat. Hormone-related components are prominent in both species as well—the auxin-efflux transporter Dw3 and the brassinosteroid-associated Dw1 in sorghum, and the gibberellin-related *FtSKIP14*/*SLY1* module together with several auxin- and ethylene-associated candidates here. The sorghum Dw genes themselves, however, have no clear orthologue among our top candidates, which is consistent with the independent domestication of a dicot pseudocereal and suggests that stem architecture in Tartary buckwheat is shaped by genes that are conserved at the level of cell-wall and hormone pathways rather than by strict one-to-one orthology.

Compared with PH and NNMS, the association signals for BNMS, SD, and TST were weaker and more dispersed across the genome. This pattern is consistent with previous studies indicating that traits related to branching and stem mechanical properties are typically controlled by multiple loci with small effects (Niu et al., 2022; Yang et al., 2024a). In particular, the lack of shared loci between BNMS and the Chr4 region suggests that branch formation may be regulated by genetic pathways distinct from those controlling main stem elongation, which is in line with the established role of auxin, cytokinin, and strigolactone signaling in axillary bud development (Schneider et al., 2019; Nahas et al., 2024). The independent genetic control of branch development provides greater breeding flexibility, enabling BNMS to be optimized while maintaining desirable PH and NNMS.

An ideal plant architecture in Tartary buckwheat is unlikely to depend on maximizing any single trait. Instead, it should result from a balanced combination of PH, NNMS, BNMS, and SD/TST. Our GWAS results suggest that accessions with moderate PH, balanced branching, and stronger stems are more likely to achieve this balance. Moderate PH can reduce lodging risk while maintaining a suitable BNMS may improve canopy structure and resource allocation, while greater SD and TST contribute to mechanical support. The genetic architecture identified here also indicates that these traits are regulated by both shared and independent loci. This provides opportunities to improve multiple architectural traits simultaneously through pyramiding favorable alleles. For example, loci affecting both PH and NNMS may enable coordinated improvement of PH and NNMS whereas loci controlling BNMS, SD, and TST could be selected independently to optimize branching and stem strength.

Several candidate genes identified in this study appear particularly promising for ideotype breeding. *TGA10* and *bZIP19* encode transcription factors that may influence multiple developmental pathways and therefore represent potential upstream regulators of plant architecture. Candidate genes related to *PGIP1* and pectin lyase are likely involved in cell-wall remodeling, which may explain why they were associated with both PH and NNMS, as changes in cell-wall properties can affect both internode elongation and organ formation. The receptor-like kinase located within the Chr4 hotspot may also participate in coordinating extracellular signaling with stem growth. In comparison, stem-related traits were associated with a different group of genes. The *FBH4* homolog identified for SD is predicted to regulate vegetative growth through transcriptional control, whereas the *PMEI* homolog associated with TST is likely involved in pectin modification and cell-wall stiffening. These genes may therefore provide useful targets for improving stem strength without substantially changing PH. Candidate genes associated with BNMS, including those related to *ABCB15*, *CYP98A3*, and *AP2/ERF*, are involved in hormone transport, secondary metabolism, and transcriptional regulation, respectively. Their distribution at independent genomic regions further suggests that branch development is genetically distinct from stem elongation, making it feasible to optimize branch number without compromising other key architectural traits.

The relatively low broad-sense heritability estimates observed for several traits in this study warrant further discussion. Tartary buckwheat is known to be highly sensitive to meteorological conditions, and temperature, altitude, and rainfall have been identified as primary drivers of its growth and development (Li et al., 2021). The three experimental environments in this study varied in altitude (1,914–2,501 m a.s.l.), mean growing-season temperature (15.25–18.05 °C), and rainfall conditions (**Table 1**), likely generating pronounced genotype × environment interactions that increased environmental variance (σ²e) relative to genetic variance (σ²g), thereby lowering H² estimates. It is also worth noting that even in F3 populations grown under relatively controlled and uniform conditions, broad-sense heritability for plant height in Tartary buckwheat was reported to be only 0.62, with narrow-sense heritability as low as 0.26 (Li et al., 2012). In a natural germplasm collection evaluated across ecologically contrasting environments, as in the present study, lower H² estimates are therefore expected. Importantly, the primary GWAS conclusions are based on BLUE values that integrate genetic signals across all three environments, and on signals consistently detected in multiple environments. The leading Chr4 signal exceeds the significance threshold by more than five orders of magnitude and is recovered for two correlated traits and in two independent datasets, demonstrating that the reliability of the main findings does not depend on the absolute magnitude of H².

Two limitations of the present work should be noted. First, lodging score and grain yield were not recorded in this panel, so the link between the traits measured here and lodging resistance is inferred from previous work rather than demonstrated directly. In Tartary buckwheat, SD, TST and PH are the principal morphological determinants of lodging: thicker stems and thicker stem walls increase stem breaking strength, whereas excessive PH and NNMS increase the lodging index and reduce grain yield (Xiang et al., 2016, 2019). SD in particular is a well-established contributor to lodging resistance and yield formation in cereals (Kashiwagi et al., 2008), and stem mechanical strength is a general target for lodging mitigation (Yang et al., 2024a). Lodging is strongly dependent on season-specific wind and rainfall and requires dedicated non-destructive scoring plots, which is why it was not included in the present multi-environment trial. Scoring lodging and grain yield in the same panel is now a priority, and will allow the Chr4 block, the SD locus on Chr1 and the TST locus on Chr8 to be tested directly for effects on lodging resistance and productivity.

Second, the association analysis used a linear regression model without principal-component or kinship covariates, so residual population stratification and cryptic relatedness are not explicitly controlled and may inflate test statistics, as reflected in the Q–Q plots for PH and NNMS in the 25HZ and BLUE datasets. The loci reported here should accordingly be regarded as candidates for validation. Three considerations nevertheless support the main conclusions: the leading Chr4 signal exceeds the significance threshold by more than five orders of magnitude and is recovered for two correlated traits and in two independent datasets; BNMS, SD and TST show little inflation; and candidate genes were prioritized on the basis of convergent functional annotation and cross-trait evidence rather than on P values alone. Re-analysis of this panel with mixed models that incorporate principal components and a kinship matrix, together with validation in independent populations, will be required to confirm the individual loci.

## 5 Conclusion

This study provides the first multi-environment genome-wide dissection of stem architecture in Tartary buckwheat. Four conclusions emerge. First, the panel of 244 accessions shows substantial and continuously distributed variation for PH, SD, BNMS, NNMS, and TST; broad-sense heritability varied among traits and environments, with lower estimates for PH, SD, and NNMS likely reflecting the high environmental sensitivity of Tartary buckwheat across contrasting field conditions, confirming that these traits are quantitatively inherited and amenable to genetic improvement. Second, a single 88-kb region on Chr4 (31.01–31.10 Mb) is the principal determinant of both PH and NNMS; it contains a co-regulated block of genes for cell-wall remodeling (*PGIP1*, a pectin lyase-like gene), vesicle trafficking (*RABA4D*) and transcriptional regulation (*TGA10*, *bZIP19*, *HAT14*), and accounts for the strong phenotypic and genetic correlation between the two traits. Third, BNMS, SD and TST are controlled by largely independent and more polygenic loci, so branching and stem mechanical strength can be selected without the pleiotropic constraints that link PH and NNMS. Fourth, comparison with sorghum indicates functional convergence on cell-wall remodeling and hormone signaling, although the specific genes are not orthologous. Taken together, these results show that an ideotype combining moderate PH, adequate NNMS, controlled BNMS and thick SD/TST, thick-walled stems is genetically attainable, and they deliver a shortlist of loci—above all the Chr4 block—for marker-assisted selection and functional validation in breeding lodging-resistant, mechanization-ready Tartary buckwheat cultivars.

## 6 Additional requirements

This study conducted the first multi-environment GWAS for five stem architectural traits in 244 Tartary buckwheat landraces. A major significant association region on Chr4 harboring coordinated cell-wall remodeling genes was identified as the primary genetic determinant of PH and NNMS, while BNMS, SD, and TST are controlled by independent genetic modules with increasingly polygenic architectures. The genetic independence among trait groups indicates that PH, BNMS, and SD/TST can be improved simultaneously without antagonistic trade-offs. The integrative composite scoring approach effectively prioritized biologically meaningful candidate genes, with the Chr4 block providing an immediate target for marker-assisted selection. These findings establish a genetic framework for optimizing stem architecture in Tartary buckwheat breeding.

## Data Availability

The raw sequencing data have been deposited in the National Genomics Data Center (NGDC: https://ngdc.cncb.ac.cn/), Genome Sequence Archive (GSA), under BioProject accession number PRJCA058783.
